# Anion Doping of Ferromagnetic Thin Films of La_0.74_Sr_0.26_MnO_3−δ_ via Topochemical Fluorination

**DOI:** 10.3390/ma11071204

**Published:** 2018-07-13

**Authors:** Parvathy Anitha Sukkurji, Alan Molinari, Christian Reitz, Ralf Witte, Christian Kübel, Venkata Sai Kiran Chakravadhanula, Robert Kruk, Oliver Clemens

**Affiliations:** 1 Fachgebiet Materialdesign durch Synthese, Institut für Materialwissenschaft, Technische Universität Darmstadt, Alarich-Weiss-Straße 2, 64287 Darmstadt, Germany; parvathy.anitha@partner.kit.edu; 2 Institut für Nanotechnologie, Karlsruher Institut für Technologie, Hermann-von-Helmholtz-Platz 1, 76344 Eggenstein-Leopoldshafen, Germany; alan.molinari@partner.kit.edu (A.M.); christian.reitz@kit.edu (C.R.); ralf.witte@kit.edu (R.W.); christian.kuebel@kit.edu (C.K.); cvskiran@kit.edu (V.S.K.C.); robert.kruk@kit.edu (R.K.); 3 Electrochemical Energy Storage, Helmholtz Institute Ulm, Helmholtzstraße 11, 89081 Ulm, Germany; 4 Karlsruhe Nano Micro Facility, Karlsruher Institut für Technologie, Hermann-von-Helmholtz-Platz 1, 76344 Eggenstein-Leopoldshafen, Germany

**Keywords:** lanthanum strontium manganite, ferromagnetism, fluorination, thin films

## Abstract

Chemical doping via insertion of ions into the lattice of a host material is a key strategy to flexibly manipulate functionalities of materials. In this work, we present a novel case study on the topotactic insertion of fluoride ions into oxygen-deficient ferromagnetic thin films of La_0.74_Sr_0.26_MnO_3−δ_ (LSMO) epitaxially grown onto single-crystal SrTiO_3_ (STO) substrates. The effect of fluorination on the film structure, composition, and magnetic properties is compared with the case of oxygen-deficient and fully-oxidized LSMO films. Although incorporation of F^−^ anions does not significantly alter the volume of the LSMO unit cell, a strong impact on the magnetic characteristics, including a remarkable suppression of Curie temperature and saturation magnetization accompanied by an increase in magnetic coercivity, was found. The change in magnetic properties can be ascribed to the disruption of the ferromagnetic exchange interactions along Mn-anion-Mn chains driven by F^−^ doping into the LSMO lattice. Our results indicate that F^−^ doping is a powerful means to effectively modify the magnetic functional properties of perovskite manganites.

## 1. Introduction

After the discovery of giant magnetoresistance [1], rare earth perovskite-type (ABX_3_) manganites with composition LnMnO_3_ (Ln = lanthanides such as La, Dy or Gd) have attracted remarkable attention due to their wide range of potential applications [1,2,3,4,5] and interesting physical, magnetic and electronic properties [6,7]. For LaMnO_3_, it was found that partial substitution of La with divalent alkaline-earth cations AE = Sr^2+^, Ca^2+^, Ba^2+^, allows transformation of the compound from a non-metallic antiferromagnet into a metallic ferromagnet with a Curie temperature (*T*_C_) close to room temperature [8]. This cation doping results in complex magnetoelectronic and structural phase diagrams of La_1−x_AE_x_MnO_3_ [9].

One of the significant features of La_1−x_Sr_x_MnO_3_ (LSMO) is that, for a composition around x = 0.25 [9], the para-ferromagnetic transition temperature, and accordingly also the insulating/metallic transition temperature, can be tailored to just below or above room temperature by relatively small variations of the doping value of x [10,11]. Since La substitution with divalent Sr cations is [9,12] directly related to an increase of the hole doping concentration, strong efforts have been devoted to studying the influence of charge carrier doping [11,12] on the magnetic and conducting properties of LSMO and other strongly-correlated oxides.

In recent years, novel routes based on X-site anion doping, such as intercalation of oxygen [13] anions, rather than conventional A-site cation doping were explored to modify the properties of perovskite-type materials. Low-temperature topotactic fluorination, implying the incorporation of monovalent F^−^ anions, was found to be [14,15,16,17] a powerful tool to change material properties of transition metals with perovskite (or perovskite-related) crystal structure by turning them into oxyfluorides. As the synthesized oxyfluorides systems are mostly metastable, low temperature topotactic fluorination routes are often required for the synthesis [18]. Although a variety of fluorination reactions [18] performed previously rely on the use of toxic F_2_ gas [16], a fairly versatile, mild and safe route exists using polymers, e.g., polyvinylidene fluoride (PVDF) [16].

This polymer-based fluorination method was previously applied to perovskite-based thin films [19,20,21,22] in order to investigate the modification of their conductivity characteristics [21]. Furthermore, such polymer approach allows for a large flexibility of the synthesis process: polymer deposition (which is a precondition for the fluorination reaction) can take place in direct contact at the film surface or within a tube furnace setup so that decomposition products (such as CH_2_CF_2_ or HF) are transported to the film surface, where they react further [22]. Remarkably, the fluorine incorporation can thus be performed at relatively low temperatures compared to bulk powders [22], most likely facilitated by the low film thicknesses and a low melting temperature (~170 °C) of the polymer with partial increased reactivity and volatility of the smaller polymer chains.

Since magnetism in LSMO and related perovskite-type manganites originates from the indirect exchange interactions [23], namely double-exchange and superexchange, between Mn ions mediated by O^2−^ anions, one could expect a significant variation of the magnetic properties of LSMO upon partial substitution of O^2−^ with F^−^ ions. In this article, we present a thin film fluorination study of epitaxially-grown films of La_1−x_Sr_x_MnO_3−δ_ (LSMO, x = 0.26). We show that the reactivity towards fluorine incorporation strongly depends on the degree of anion vacancies within the lattice and can lead to remarkable changes of the magnetic properties.

## 2. Materials and Methods

The LSMO films were grown on (001)-oriented single-crystalline SrTiO_3_ (STO) substrates using a large-distance magnetron sputtering (LDMS) chamber featuring an unconventionally-large target-to-substrate separation of 285 mm. STO was chosen as a substrate, because it has a perovskite structure and a low lattice mismatch with LSMO, and both conditions can facilitate the epitaxial growth of LSMO onto STO. Prior to the deposition process, the surface quality of the STO substrates was analyzed via atomic force microscopy (AFM, Bruker, Madison, WI, USA), which revealed a flat morphology with a root mean square roughness of about 0.2 nm. For all depositions, an LSMO ceramic disc of 3 inches in diameter was used as the sputtering target. The LSMO samples were prepared at a temperature of about 800 °C and a pressure of 0.018 mbar in Ar/O_2_ = 3/2 gas mixture. A sputtering power of 200 W led to a deposition rate of 0.045 Å/s, which was monitored with a quartz crystal microbalance. As we were aiming to produce LSMO films with inherent oxygen vacancies, after completing the deposition the samples were cooled to room temperature at a rate of 10 K/min under a relatively-low oxygen pressure of 0.08 mbar. Further details about the LSMO growth conditions can be found elsewhere [24].

The obtained La_0.74_Sr_0.26_MnO_3−__δ_ films with inherent oxygen vacancies were subjected to various heat treatments and fluorination processes in order to be able to study the influence of different anion-incorporating conditions on the film characteristics. Those treatments are summarized in Table 1. All heat treatments were conducted in a Carbolite 1260 tubular furnace, which can be operated under different gas atmospheres. The PVDF-based fluorination method is similar to the one previously reported by Katayama et al. [22], and is based on the low melting point of the polymer of ~170 °C, which allows for partial volatilization of the smaller polymer chains. The film and PVDF were kept in separate ceramic alumina boats to prevent direct contact between them, thus resulting in milder fluorination conditions and to avoid decomposition of the fluorinated films [25].

The surface roughness of the samples was characterized by tapping-mode AFM under ambient conditions. High resolution X-ray diffraction (HRXRD, Bruker, Karlsruhe, Germany) measurements were carried out using a Bruker D8 Advance thin film diffractometer in *θ*–2*θ* configuration with Cu-Kα1 (λ = 1.540596 Å) radiation in order to analyze the phase evolution of the LSMO films treated with different reaction conditions. Analysis of the out-plane lattice parameter of the various films was performed by using the Pawley method as implemented in the TOPAS software package [26], after the instrumental broadening was determined via a Fundamental Parameters method [27]. This estimation is basically relying on the evaluation of the Bragg position of the film peak, neglecting interference effects as reported by Pesquera et al. [28], which is small for film thicknesses above 20 nm.

The magnetic characterization was performed using a Quantum Design MPMS SQUID Magnetometer (Quantum Design, San Diego, CA, USA). Field cooled (FC) curves were measured in the 5 to 400 K temperature range with an external magnetic field of 500 Oe applied parallel to the in-plane film direction to determine the value of *T*_C_. Magnetic hysteresis loops carried out at the temperature of 5 K allowed to analyze the saturation magnetization and the magnetic coercivity.

Transmission electron microscopy (TEM, FEI Company, Portland, OR, USA) was performed within an aberration (image) corrected FEI Titan 80–300 operated at an acceleration voltage of 300 kV. Elemental distributions were imaged by energy filtered TEM using the “3-windows approach” with a Gatan Tridium 863 energy filter. The study was performed on cross-sections of the LSMO thin film specimens prepared by focused ion beam milling (FIB, FEI Company, Portland, USA using a Strata 400 S. In order to protect the film surface during milling process a thin gold coating was deposited on the film via sputtering prior to the FIB preparation.

## 3. Results and Discussion

### 3.1. Morphology, Composition and Structure of the Films

**Figure 1:**
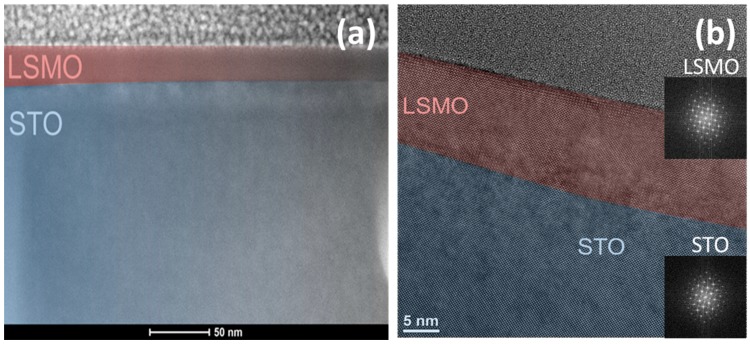
(**a**) Cross-sectional high-angle annular dark field (HAADF) scanning transmission electron microscopy (STEM) image and (**b**) High-resolution TEM image of the fluorinated LSMO film (LSMO_F).

The thickness of the fabricated LSMO films was about 20 nm, as determined by means of X-ray reflectometry and TEM imaging (see Figure 1). The growth of LSMO on STO (001) was found to be epitaxial, in agreement with previous reports [11,29]. The epitaxial nature of the LSMO films was found to be maintained after fluorination, confirming that the chosen fluorination conditions are suitable to avoid decomposition into lanthanum oxide fluoride or strontium fluoride, which are well-known decomposition products of metastable oxyfluorides [30].

The AFM analysis revealed a smooth surface morphology of the as-grown LSMO films with values of root mean square (rms) roughness as small as 0.24 nm (not shown here). After fluorination (LSMO_F), only a slight increase of the rms roughness (~0.7 nm) was observed, thus confirming that the fluorinating agent (PVDF) does not cause a substantial alteration of the LSMO surface topography due to decomposition and/or polymer deposition.

**Figure 2:**
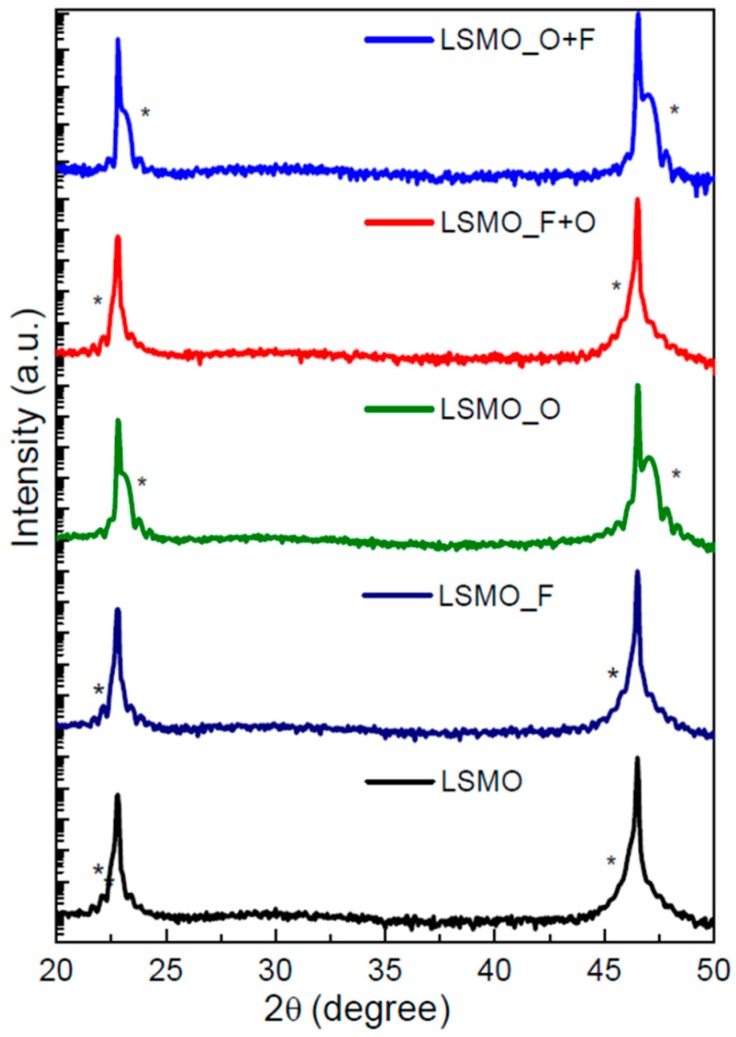
X-ray diffractograms of the treated LSMO thin films (where * denotes the LSMO film peaks).

HRXRD patterns revealed that the reflections of as-grown and topochemically-treated LSMO films follow the same (001)-orientation of the STO substrate peaks (see Figure 2) with the absence of impurity phases (e.g., LaOF or SrF_2_). The out-of-plane lattice parameter of the different films is summarized in Table 2. In case of the as-grown sample, film and substrate peaks mainly overlap on top of each other. This behavior is compatible with the presence of oxygen deficiencies, which are known to cause an expansion of the LSMO unit cell volume along the out-of-plane direction [11].

Oxidation of the as-grown samples (LSMO_O) results in a significant shift of the LSMO peaks towards higher angles, which is related to the shrinkage of the LSMO unit cell volume owing to the smaller size of the Mn^4+^ ion as compared to Mn^3+^ [25]. The contraction of the out-of-plane lattice parameter upon oxidation is not only known for manganites [31], but also for other perovskite-type compounds [32,33].

In contrast, the fluorination of the as-grown film (LSMO_F) does not strongly alter the out-of-plane lattice parameter of LSMO. In this respect, the reader should be aware that fluorination reactions of perovskite materials (see e.g., [18] for a review) can be classified in three different reaction mechanism, where the detailed reaction pathway chosen depends on the material system and fluorination agent in use:

(a) Reductive fluorination: One fluoride ion replaces one oxygen ion, accompanied by a reduction of the transition metal oxidation state
(b) Substitutive fluorination: Two Fluoride ions replace one oxygen ion and fill a vacancy under maintenance of the transition metal oxidation state
(c) Oxidative fluorination: One Fluoride ion fills a vacancy, accompanied by an increase of the transition metal oxidation state

Usually, the cell volume per formula unit (V_f.u._) can give a strong indication for which fluorination mechanism has taken place. This is explained by the empirical finding that the volume per formula unit mainly depends on the transition metal oxidation state and not on the detailed composition of oxygen and fluoride ions as well as vacancies of the anion sublattice [18,32,33,34] the higher the transition metal oxidation state, the smaller its ionic radius and the lower the volume per formula unit. This can be exemplified by the following: for the fluorination of SrFe^3+^O_2.5_ and BaFe^3+^O_2.5_ under formation SrFe^3+^O_2_F [32] and BaFe^3+^O_2_F [32,35] (0 ≤ 2x ≤ 1), i.e., corresponding to mechanism (b), the average change of the cubic (pseudocubic) lattice parameter is as low as 0.5% for the two end members (ΔV~1.5%). In contrast, the fluorination of SrFeO_3_ to SrFeO_2_F (mechanism (a) goes with a strong increase of the unit cell (ΔV~+6%) [32,36], whereas the fluorination of brownmillerite compounds AMO_2.5_ to AMO_2.5_F_0.5_ (mechanism (c)) is accompanied by a strong shrinkage of the unit cell [33,34]. The maintenance of the unit cell volume therefore strongly indicates a substitutive fluorination according to mechanism (b).

**Figure 3:**
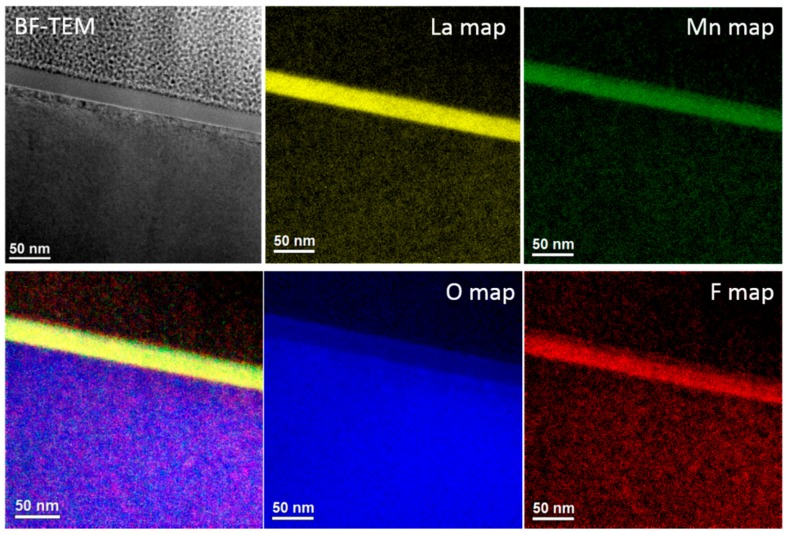
Energy filtered TEM elemental maps of the LSMO_F film showing fluorine incorporation into the LSMO layer.

In order to confirm fluorination of the film and in order to verify if the small influence of fluorination on the LSMO unit cell volume may be caused by incorporation of F^−^ anions predominantly at the film surface (i.e., without affecting the bulk crystal structure of LSMO), a cross-section of sample LSMO_F was analyzed by means of elemental mapping via energy filtered TEM (EFTEM), see Figure 3. Indeed, it was found that fluorine incorporation takes place throughout the entire LSMO film thickness. In contrast, no fluorination of the STO substrate was seen, in agreement with the fact that STO does not possess significant amounts of oxygen deficiencies. In addition, a reduced oxygen content of the LSMO film compared to the STO substrate was found. Therefore, the EFTEM analysis demonstrates that fluorination affects the main volume of the LSMO film. Additional details on the EFTEM investigation are described in the Supplementary Information (also see Figure S1 there).

The importance of the presence of anion vacancies, which are required for a substitutive fluorination according to mechanism (b), was further examined by reacting differently treated films. Application of fluorination treatment to an oxidized film (LSMO_O+F) resulted in a mainly unreactive response with the absence of significant changes of the LSMO lattice parameter (and in agreement with the absence of changes of magnetic properties as discussed in Section 3.2). Similarly, attempts to oxidize a fluorinated film (LSMO_F+O) did not show a prominent reduction of the out-of-plane lattice parameter, thus denoting a low sensitivity towards oxidation. These findings prove that the oxidation and fluorination of the as-grown LSMO films result in almost completely filling of the anion sublattice of the perovskite structure.

We, therefore, summarize the reaction of as-grown oxygen deficient La_1−x_Sr_x_MnO_3−y_ with HF (from PVDF decomposition) and O_2_ by the following equations, again highlighting the importance of the presence of anion vacancies V_O_^••^ for the fluorine incorporation:

(LSMO_F): La_1−x_Sr_x_**Mn^3+^_1−x+2y_Mn^4+^_x−2y_**O_3−y_ + 2y HF ➔ La_1−x_Sr_x_**Mn^3+^_1−x+2y_Mn^4+^_x−2y_**O_3−2y_F_2y_ + y H_2_O
(LSMO_O): La_1−x_Sr_x_**Mn^3+^_1−x+2y_Mn^4+^_x−2y_**O_3−y_ + ^y^/_2_ O_2_ ➔ La_1−x_Sr_x_**Mn^3+^_1−x_Mn^4+^_x_**O_3_
(LSMO_F+O): La_1−x_Sr_x_**Mn^3+^_1−x+2y_Mn^4+^_x−2y_**O_3−2y_F_2y_ + O_2_ ➔ no reaction
(LSMO_O+F): La_1−x_Sr_x_**Mn^3+^_1−x_Mn^4+^_x_**O_3_ + HF ➔ no reaction

### 3.2. Magnetic Characterization

**Figure 4:**
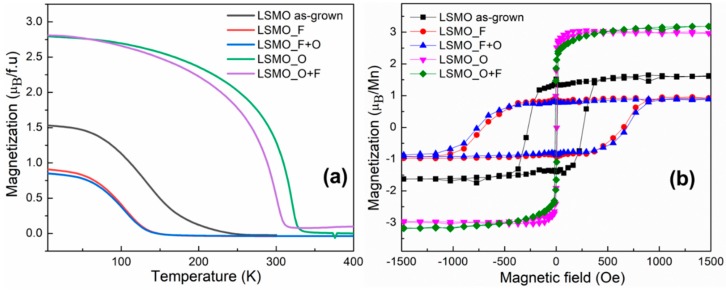
(**a**) Field-cooled magnetization measurements and (**b**) Field-sweep hysteresis loops of the investigated LSMO films measured at 5 K. The magnetization per Mn ion was estimated from the film thickness and is in fair agreement with previous findings for as-grown and oxidized films [11,23].

Figure 4a shows the magnetic FC curves of the as-grown LSMO and the films treated under different topochemical reactions. Concerning the as-grown LSMO film, a reduced value of *T*c~135 K compared to bulk LSMO and a rather smeared out para/ferromagnetic transition is found, in agreement with the behavior encountered in oxygen-deficient LSMO thin films [37]. As expected, upon oxidation the film (LSMO_O) undergoes a remarkable increase of *T*c up to about 320 K. Differently, in case of incorporation of fluoride ions (LSMO_F), the magnetic transition temperature decreases down to 100 K. Processing of LSMO_O and LSMO_F with a sequential fluorination/oxidation treatment, i.e., LSMO_O+F and LSMO_F+O respectively, did not bring any substantial modification of the measured FC curves.

Further insights into the influence of different conditions of anion incorporation on the magnetic properties were gained from the analysis of low-temperature magnetic hysteresis loops (see Figure 4b). Starting from an as-grown film (*M*s~1.5 µ_B_/Mn., *H*c~270 Oe), oxidation treatment led to an increase in saturation magnetization (*M*s~3 µ_B_/Mn.) and decrease in coercive field (*H*c~8 Oe), whereas fluorination produced a much lower magnetic moment (*M*s~0.9 µ_B_/Mn) and a broader hysteresis loop (*H*c~760 Oe). It is worth to notice that fluorinated LSMO is magnetically harder than oxidized LSMO with a difference in magnetic coercivity of almost two orders of magnitude. Additionally, the measurements confirmed that an a-posteriori change of the nearly-completely filled anion lattices of the LSMO_O/LSMO_F films is not possible at the chosen reaction conditions, since no prominent changes of magnetization curves could be found for the LSMO_O+F and LSMO_F+O films. However, it is acknowledged that such changes might be induced in different reaction conditions (higher temperature, higher polymer excess, reaction atmosphere).

The obtained values of Curie temperature *T*_C_, saturation magnetization *M*_S_, and coercive field *H*_C_ are listed in Table 3. According to the experimental results, incorporation of either oxygen or fluorine anions into the oxygen-deficient lattice of LSMO have a remarkable impact on the magnetic characteristics. The magnitude of the change in *T*_C_, *M*_S_, and *H*_C_ indicate that anion intercalation affects a large portion of the LSMO volume, thus corroborating the results of the microstructural analysis.

In a conventional scenario, the magnetic properties of manganites are determined by the exchange interactions along Mn-O-Mn bonds due to the overlap of the manganese d-orbitals with the p-orbitals of the bridging anions [32]. Ferromagnetic coupling between Mn^3+/4+^ ions originates from double-exchange mechanism, whereas antiferromagnetic interactions are promoted between Mn^3+/3+^ or Mn^4+/4+^ via superexchange mechanism.

The fact that as-grown oxygen-deficient films reveal a suppressed magnetization and Curie temperature is compatible with an excess of Mn^3+^ and missing oxygen bonds. With the insertion of oxygen anions, Mn-O-Mn bonds are restored and the Mn^3+/4+^ balance increases owing to the covalency of O^2−^, which amounts to doping of the LSMO unit cell with two holes. As a consequence, oxidized films present enhanced *T*c and *M*s, in agreement with the values expected from the LSMO magnetic phase diagram [9] for a composition of Sr = 0.26.

To our knowledge, the effect of the substitutive fluorination on the magnetic properties of manganites has not been theoretically investigated yet. Experimental studies only exist for the oxidative fluorination of Ruddlesden-Popper type manganites [38,39], which cannot be easily compared to the system studied in this work. Here, we suggest possible scenarios for the interpretation of the experimental data.

Apparently, the very different magnetic behavior observed after fluorination (LSMO_F) and oxidation (LSMO_O) may be primarily attributed to the different balance in Mn^3+/4+^ ratio, driven by the fact that F^−^ insertion amounts to doping the LSMO unit cell with only one hole. In this respect, fluorinated LSMO should give a similar magnetic response comparing to as-grown LSMO films, since fluorination follows a substitutive mechanism and the Mn^3+/4+^ ratio is maintained (see reaction equation “LSMO_F” given in Section 3.1). Nonetheless, the experimental results clearly evidence a remarkable decrease of both *T*c and *M*s upon F^−^ incorporation, thus indicating that other mechanisms must come into play.

One point that, in accordance with the Goodenough–Kanamori rules [5], could justify the unexpected magnetic response upon fluorination is the different nature of the Mn-X-Mn chemical bonds, which are predominantly covalent in case of X = O^2−^, and more ionic for X = F^−^. In this regard, F^−^ doping may trigger antiferromagnetic coupling or extinguish any exchange interaction between adjacent Mn ions. This observation is in principle agreement with the recently reported weakening of Fe-F-Fe superexchange interactions as compared to Fe-O-Fe for the series LaFe^3+^O_3_F_0_ (T_N_~750 K), (Sr/Ba/Pb) Fe^3+^O_2_F_1_ (T_N_ = 650–700 K) and AgFe^3+^O_1_F_2_ (T_N_~500 K) [40], as well as the weakening of Ni-F-F-Ni superexchange interactions in La_2_Ni^2+^O_3_F_2_ (T_N_ = 49 K) as compared to Ni-O-O-Ni in La_2_NiO_4_ (T_N_ = 330 K) [14].

Another factor to keep in mind, related to a figure of merit known as tolerance factor *Γ* [31], is the alteration of bond lengths and angles caused by the different ionic radius of fluorine with respect to oxygen; however, this effect is comparatively small for the substitutive fluorination as compared to oxidative or reductive fluorination mechanism (see Figure S2 within the Supplementary Information for further details) and might, therefore, be of subordinate importance. In addition, the spatial disorder caused by the presence of both Mn-O-Mn and Mn-F-Mn chains (with their possible mutual relation still being unknown) is another disruptive factor, which could give rise to a further suppression of the ferromagnetic interactions. Finally, the possible involvement of other phenomena occurring in manganites, such as magnetic phase separation [41], vacancy ordering mechanisms and the magnetic dead layer [42] at the LSMO/STO interface, cannot be ruled out and requires further in-depth investigation.

## 4. Conclusions and Outlook

In this work, we have compared the influence of topochemical oxidation as well as substitution of divalent O^2−^ with monovalent F^−^ anions on the structural and magnetic properties of oxygen-deficient thin films of LSMO epitaxially grown on STO substrate. Both oxidation and fluorination processes involve anion incorporation into the bulk of the LSMO films, although no remarkable expansion or shrinkage of the LSMO unit cell volume occurs upon F^−^ doping. Concerning the magnetic properties, the processes of oxidation and fluorination have completely different effects on the as-grown LSMO films. The former makes LSMO a stronger and softer ferromagnet, whereas the latter leads to an increase of the magnetic coercivity at the expense of the Curie temperature and magnetization saturation. As to the physical origins of the strong suppression of the ferromagnetic order, further theoretical studies are necessary to unveil the complex mechanisms controlling the magnetic exchange interactions in LSMO upon F^−^ doping.

This work suggests that topochemical fluorination can offer an alternative route to conventional doping approaches based on divalent cation or oxygen anion incorporation to manipulate the functional properties of strongly correlated perovskite manganites. Since fluorine ions are more mobile than oxygen ions [43], their reversible insertion and extraction may be realized by means of electrochemical methods [34,44]. Such methods, e.g., by using electrochemical oxidative fluorine insertion (AMnO_3−y_ → AMnO_3−y_F_y_), or reductive fluorine deintercalation (AMnO_3−y_F_y_ → AMnO_3−y_), shall open new opportunities for tuning material properties.

## Figures and Tables

**Table 1:** Synthesis conditions for the different La_0.74_Sr_0.26_MnO_3−δ_ (LSMO)-based films reported in this study.

Sample Label	Reaction Conditions
LSMO	As-grown LSMO film
LSMO_F	LSMO film fluorinated at 180 °C for 12 h in argon
LSMO_O	LSMO film oxidized at 900 °C for 1 h in air
LSMO_O+F	LSMO film oxidized at 900 °C in air for 1 h, followed by fluorination at 180 °C in argon carried out for 12 h
LSMO_F+O	LSMO film fluorinated at 180 °C for 12 h, followed by oxidation at 240 °C overnight in air (here reduced oxidation temperature is required to account for the metastable nature of the oxyfluoride compound)

**Table 2:** Analysis of out-of-plane lattice parameters of the various LSMO films (for detailed preparation conditions, see Table 1). Numerical errors for the determined parameters from the Pawley fit are in the order of ±0.001 Å, the tolerance from neglecting X-ray interference effects [28] was estimated to be below ±0.005 Å for films of 20–25 nm thickness.

Thin Film Label	Out-of-Plane Lattice Parameter of the Film (Å)
LSMO As-grown	3.925
LSMO_F	3.920
LSMO_F+O	3.920
LSMO_O	3.860
LSMO_O+F	3.865
STO substrate	3.905

**Table 3:** Values of saturation magnetization (*M*s), Curie temperature (*T*c) and coercive field (*H*c) as obtained from the magnetization measurements shown in Figure 4. Experimental errors are within 10% accuracy.

Type of Film	Curie Temperature *T*_C_ (K)	Saturation Magnetization *M*_S_ at 5 K (μB per Mn ion)	Coercive Field *H*_C_ (Oe)
LSMO as-grown	135.1	1.5	271.5
LSMO_O	320.0	3.0	7.8
LSMO_O+F	301.1	3.2	9.2
LSMO_F	103.4	0.9	704.3
LSMO_F+O	103.2	0.8	760.5

## Supplementary Materials

The following are available online at http://www.mdpi.com/1996-1944/11/7/1204/s1, Figure S1: HRTEM image of the LSMO_F film imaged in [001] zone axis with FFTs corresponding to different parts of the film, Figure S2: Dependence of Goldschmidt’s tolerance factor for the different types of fluorination reactions under neglectance of ordering effects for anion deficient systems.
