# Supplementary material for: Anion Doping of Ferromagnetic Thin Films of La0.74Sr0.26MnO3−δ via Topochemical Fluorination
Source: Materials (Basel). 2018 Jul 13;11(7):1204. doi: 10.3390/ma11071204 (PMC6073400; doi:10.3390/ma11071204)
Supplement: Supplementary file 1 [file materials-11-01204-s001.pdf]

Supporting Information

Anion Doping of Ferromagnetic Thin Films of $La_{0.74}Sr_{0.26}MnO_{3-\delta}$ via Topochemical Fluorination

Parvathy Anitha Sukkurji[a,b,c], Alan Molinari[b], Christian Reitz[b], Ralf Witte[b], Christian Kübel[b,d,e], Venkata Sai Kiran Chakravadhanula[b,d], Robert Kruk[b], Oliver Clemens[a,b,*]

[a] Technische Universität Darmstadt, Institut für Materialwissenschaft, Fachgebiet Materialdesign durch Synthese, Alarich-Weiss-Straße 2, 64287 Darmstadt, Germany.

[b] Karlsruher Institut für Technologie, Institut für Nanotechnologie, Hermann-von-Helmholtz-Platz 1, 76344 Eggenstein-Leopoldshafen, Germany.

[c] Indian Institute of Technology Madras, Department of Engineering Design, 600036 Chennai, India.

[d] Helmholtz Institute Ulm — Electrochemical Energy Storage, Helmholtzstraße 11, 89081 Ulm, Germany.

[e] Karlsruher Institut für Technologie, Karlsruhe Nano Micro Facility, Hermann-von-Helmholtz-Platz 1, 76344 Eggenstein-Leopoldshafen, Germany.

* Corresponding Author

Fax: +49 6151 16 20965

E-Mail: oliver.clemens@md.tu-darmstadt.de

EFTEM analysis

EFTEM also indicates that the top layer of the LSMO film appears to be poorer in fluorine compared to the main part of the film closer to the STO substrate. At the same time, the oxygen content of the top-layer of the film is found to be increased. Investigating the structure further by HRTEM (see Figure S 1), a small change of the (XY0) reflection intensities with X+Y odd was found, which could indicate slight structural changes within the top layer (e. g., from symmetry of composition). The strain state of the film is of importance for topochemical reactions, which can enhance [30,31] or decrease [17] during the reaction depending on the chemically incorporated species. As indicated from the analysis of the out-of-plane lattice parameters, the epitaxial strain is lower for LSMO_F films compared to the LSMO_O films, and this might explain why an enrichment of fluorine species takes place closer to the STO substrate.

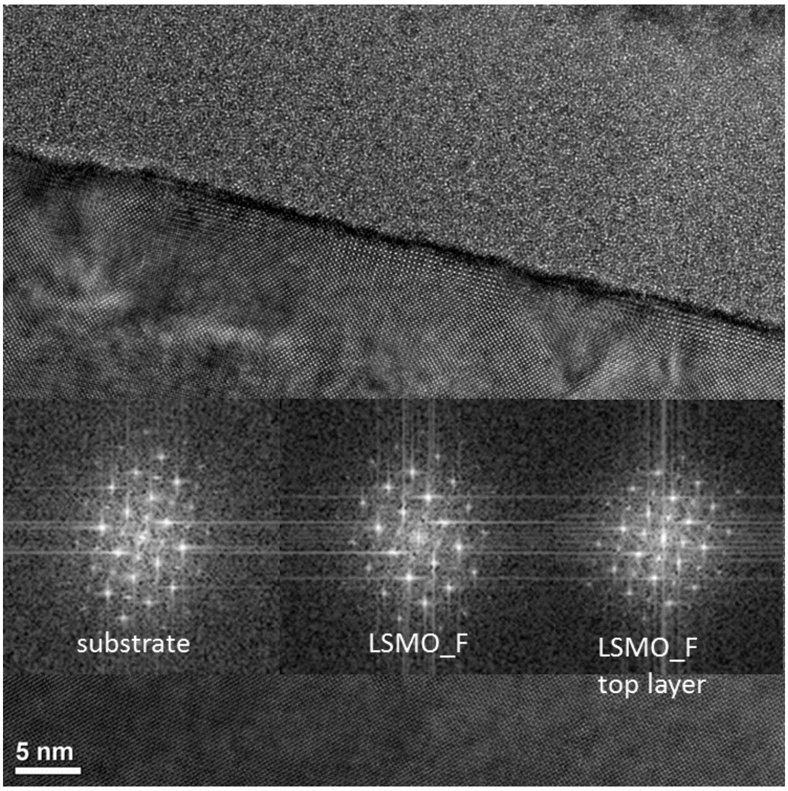

Figure S 1. HRTEM image of the LSMO_F film imaged in [001] zone axis with FFTs corresponding to different parts of the film.

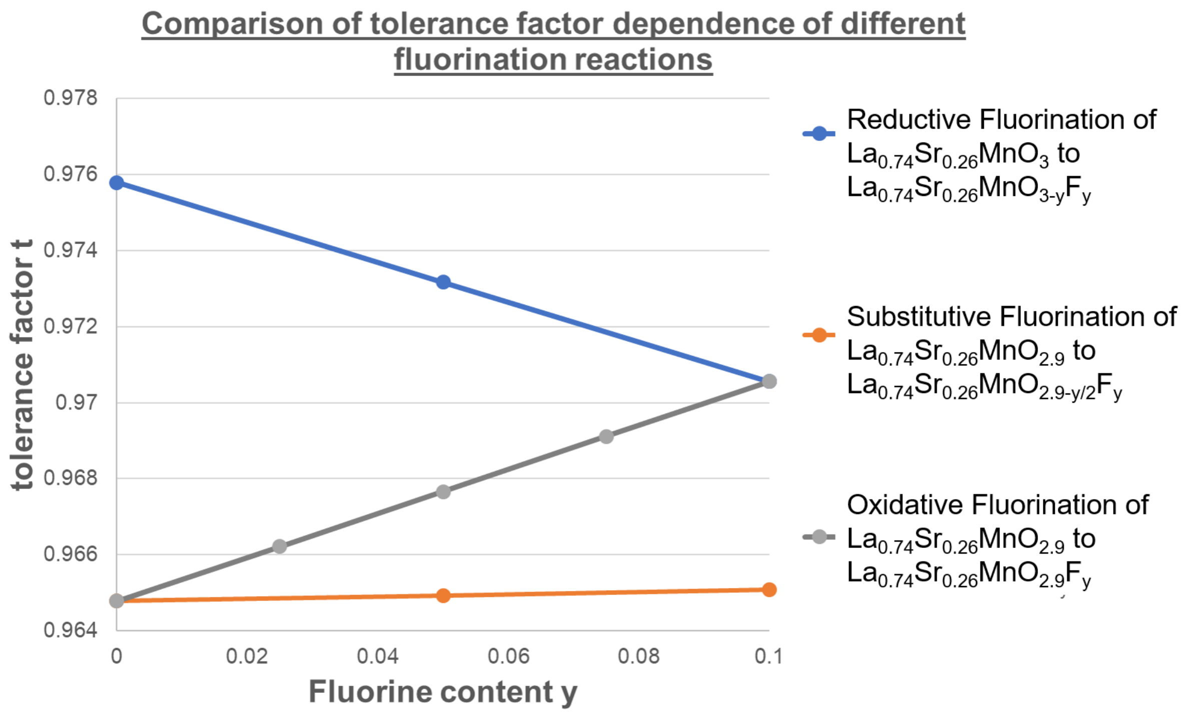

Figure S 2. Dependence of Goldschmidt's tolerance factor for the different types of fluorination reactions under neglectance of ordering effects for anion deficient systems.
